# Supplementary material for: Analytical Performances of the Novel i-STAT Alinity Point-of-Care Analyzer
Source: Diagnostics (Basel). 2023 Jan 13;13(2):297. doi: 10.3390/diagnostics13020297 (PMC9857650; doi:10.3390/diagnostics13020297)
Supplement: Supplementary file 1 [file diagnostics-13-00297-s001.zip › diagnostics-2076036-supplementary.pdf]

Supplementary Materials

Analytical Performances of the Novel i-STAT Alinity Point-of-Care Analyzer

Romaric Larcher [1,2,*], Maxence Lottelier [3], Stephanie Badiou [1], Anne-Marie Dupuy [3], Anne-Sophie Bargnoux [1] and Jean-Paul Cristol [1]

[1] Biochemistry and Hormonology Department, Montpellier University Hospital, PhyMedExp (Physiology and Experimental Medicine), INSERM (French Institute of Health and Medical Research), CNRS (French National Centre for Scientific Research), University of Montpellier, 34000 Montpellier, France
[2] Infectious and Tropical Diseases Department, Nimes University Hospital, 30000 Nimes, France
[3] Biochemistry and Hormonology Department, Montpellier University Hospital, 34000 Montpellier, France
* Correspondence: romaric.larcher@chu-nimes.fr; Tel.: +33-466-68-41-49

Table S1. Review of the existing literature on the analytical performance of various Point-of-Care (POC) analyzers

Analyzer	Test	Parameter	Mean	CV (%)
Abbott i STAT 1 or Abbott i STAT Alinity	Cartridge 6+: *Measured* Na, K, Cl, Glu, BUN/Urea, Hct.	pH	7.065 / 7.649	0.05 /0.06
		PCO2 (mmHg)	59.0 / 23.2	1.8 / 2.2
		PO2 (mmHg)	85 / 136	2.0 / 1.7
Hand held analyzers	Cartridge CG4+: *Measured* pH, PO2, PCO2, Lactate.	Lactate (mmol/L)	6.73 / 0.74	0.8 / 2.0
		Na (mmol/L)	123 / 159	0.3 / 0.5
	Cartridge CG8+: *Measured* Na, K, iCa, Glu, Hct, pH, PO2, PCO2.	K (mmol/L)	2.9 / 6.2	1.4 / 0.8
		Cl (mmol/L)	74 / 115	0.6 / 0.6
		iCa (mmol/L)	0.90 / 1.58	0.8 /0.8
		Glu (mmol/L)	14.4 / 2.1	0.4 / 2.1
	Cartridge CHEM8+: *Measured* Na, K, Cl, iCa, Glu, Urea, Crea, Hct, TC02.	Urea (mmol/L)	20.4 / 2.1	2.1 / 4.3
		Creat (µmol/L)	333 / 35	2.0 / 5.2
		Hematocrit (%)	22 / 55	1.0 / 0.9
	Cartridge Crea: *Measured* Crea.			
	Cartridge E3+: *Measured* Na, K, Hct.			
	Cartridge EC4+: *Measured* Na, K, Glu, Hct.			
	Cartridge EC8+: *Measured* Na, K, Cl, Glu, BUN, Hct, pH, PO2, PCO2.			

	Cartridge EG6+: *Measured* Na, K, Hct, pH, PO2, PCO2.<br>Cartridge EG7+: *Measured* Na, K, iCa, Hct, pH, PO2, PCO2.<br>Cartridge G: *Measured* Glu.<br>Cartridge G3+: *Measured* pH, PO2, PCO2.			
	Cartridge PT/INR: *Measured* Prothrombin time; *Calculated*: International normalized ratio	PT	16.5 / 28.7	2.6 / 6.3
		INR	1.4 / 2.5	3.7 / 3.7
Nova Stat Profile Prime<br><br>Bench top analyzer	Measured: pH, PCO2, PO2, sO2, lactate, O_2Hb, COHb, MetHb, HHb, HbF, Hct, Hb, Na, K, Cl, iCa, iMg, urea, Creat, Glu, tBil	pH	7.20 / 7.39 / 7.62	0.01 / 0.02 / 0.00
		PCO2 (mmHg)	57.8 / 40.9 / 19.7	5.83 / 5.43 / 4.12
		PO2 (mmHg)	54.9 / 91.2 / 128	2.96 / 2.77 / 3.61
		Lactate (mmol/L)	2.0 / 6.9	0.06 / 1.62
		Na (mmol/L)	141.2 / 116.3	0.11 / 0.21
		K (mmol/L)	3.9 / 6.2	0.13 / 0.17
		Cl (mmol/L)	128.2 / 99	1.18 / 0.65
		iCa (mmol/L)	1.1 / 1.5	0.42 / 0.04
		Glu (mmol/L)	4.5 / 15.8	0.05 / 1.62
		Urea (mmol/L)	7.3 / 23.1	0.92 / 1.84
		Creat (μmol/L)	84.3 / 583.6	3.97 / 2.43
		Hb (g/dL)	14.7 / 8.6	0.90 / 0.81
Nova Stat Sensor creatinine	*Measured:* Crea	Creat (μmol/L)	80.9 / 496.5	7.9 / 7.2
Nova Stat Strip Lactate	*Measured:* Lactate	Lactate (mmol/L)	0.9 / 6.4	8.2 / 6.8
Radiometer ABL90 FLEX<br><br>Bench top analyzer	Measured: pH, PCO2, PO2, sO2, lactate, O_2Hb, COHb, MetHb, HHb, HbF, Hb, Na, K, Cl, iCa, Glu, urea, Crea, tBil	pH	6.77 / 7.15 / 7.49	0.83 / 0.56 / 0.88
		PCO2 (mmHg)	20.2 / 28.4 / 66.0	1.50 / 1.07 / 1.16
		PO2 (mmHg)	17.5 / 188.9 / 302.1	5.23 / 0.63 / 0.33
		Lactate (mmol/L)	3.5 / 7.5	0.80 / 1.30
		Na (mmol/L)	112.2 / 135.8 / 171.1	0.22 / 0.26 / 0.18
		K (mmol/L)		1.45 / 0.53 / 0.17
		iCa (mmol/L)	1.74 / 3.73 / 6.51	1.55 / 0.47 / 0.31
		Glu (mmol/L)	0.31 / 0.81 / 1.54	1.32 / 0.70
		Urea (mmol/L)	6.8 / 14.6	1.4 / 0.8 / 1.9
		Creat (μmol/L)	4.9 / 14.4 / 30.5	2.1 / 1.9 / 1.5
		Hb (g/dL)	79 / 182 / 573	0.27 / 0.19
			12.9 / 19.3	

Roche Cobas b 123<br><br>Bench top analyzer	Measured: pH, PCO2, PO2, sO2, lactate, O_2Hb, COHb, MetHb, HHb, Hb, Na, K, Cl, iCa, Glu, tBil	pH	7.14 / 7.39 / 7.54	0.05 / 0.06 / 0.04
		PCO2 (mmHg)	24.3 / 40.3 / 65.2	0.87 / 0.84 / 1.36
		PO2 (mmHg)	62.4 / 98.7 / 145.4	6.07 / 3.16 / 1.95
		Lactate (mmol/L)	1.7 / 3.2 / 10.2	1.31 / 1.73 / 0.94
		K (mmol/L)	3.0 / 4.72 / 7.02	0.52 / 0.50 / 0.52
		iCa (mmol/L)	2.70 / 5.20 / 6.85	0.73 / 0.53 / 0.42
		Glu (mmol/L)	2.5 / 6.2 / 23.5	1.22 / 1.29 / 2.53
		Hb (g/dL)	7.5 / 11.9 / 19.7	1.52 / 1.42 / 1.37
Roche CoaguCheck Pro II	Measured: PT; *Calculated*: INR	INR	1.28 / 2.94	3.28 / 4.47
Siemens Rapid Point<br><br>Bench top analyzer	Measured: pH, PCO2, PO2, sO2, lactate, O_2Hb, COHb, MetHb, HHb, Hb, Na, K, Cl, iCa, Glu, tBil	pH	7.1 / 7.32 / 7.49	0.09 / 0.05 / 0.004
		PCO2 (mmHg)	78.7 / 43.3 / 25.0	6.07 /3.3 / 3.4
		PO2 (mmHg)	139.7 / 103.1 / 48.1	1.8 / 2.26 / 7.8
		Lactate (mmol/L)		0.7 / 0.8 / 1.21
		Na (mmol/L)	112.2 / 132.9 /	0.5 / 0.65 / 0.84
		K (mmol/L)	152.8	1.09 / 0.80 / 0.7
		Cl (mmol/L)	3.1 / 4.97 / 6.56	1.16 / 0.77 / 1.64
		iCa (mmol/L)	74.7 / 98.1 / 117.9	1.77 / 2.32 / 4.07
		Glu (mmol/L)	1.49 / 1.16 / 0.79	1.02 /1.15 / 1.94
		Hb (g/dL)	10.9 / 5.1 / 2.6	
			17.8 / 13.8 / 7.7	
Siemens epoc® Blood Analysis System<br><br>Hand held analyzer	Measured: pH, PCO2, PO2, TCO2, lactate, Hct, Na, K, Cl, iCa, urea, Crea, Glu.	pH	7.01 / 7.72	0.16 / 0.16
		PCO2 (mmHg)	70 / 22	1.1 / 3.62
		PO2 (mmHg)	67.5 : 189.5	3.75 / 3.85
		Lactate (mmol/L)	0.82 / 5.78	5.39 / 6.78
		Na (mmol/L)	115 / 162	0.72 / 0.49
		K (mmol/L)	2.1 / 5.8	0.0 / 1.16
		Cl (mmol/L)	79.6 / 113.4	1.18 / 1.77
		iCa (mmol/L)	1.54 / 0.64	1.52 2.52
		Glu (mmol/L)	1.9 / 14.6	3.73 / 2.50
		Urea (mmol/L)	18.7 / 1.7	1.97 / 2.89
		Creat (µmol/L)	69 / 340	5.6 / 2.9
		Hematocrit (%)	25 / 44	2.28 / 2;61
Siemens Xprecia Stride™ Coagulation Analyzer<br><br>Hand held analyzer	*Measured* Prothrombin time; *Calculated*: International normalized ratio	INR	1-2 / 2-3	2.8 / 3
Werfen GEM 5000<br><br>Bench top analyzer	Measured: pH, PCO2, PO2, sO2, lactate, O_2Hb, COHb, MetHb, HHb, Hb, Na, K, Cl, iCa, Glu, tBil	pH	7.13 / 7.37 / 7.58	0.1 / 0.09 / 0.04
		PCO2 (mmHg)	14 / 37.7 / 90	0 / 2.5 / 2.3
		PO2 (mmHg)	31.8 / 87 / 349.5	5.7 / 3.0 / 4.0
		Lactate (mmol/L)	0.8 : 1.6 / 2.3	4.3 / 3.7 / 1.0

		Na (mmol/L)	124.1 / 139.6 / 154	0.4 / 0.6 / 0.3
		K (mmol/L)	2.4 / 4.6 / 7.7	0.0 / 0.6 / 0.8
		Cl (mmol/L)	85.6 / 108.5 / 141.8	0.7 / 0.5 / 0.4
		iCa (mmol/L)	0.64 / 1.16 / 1.59	0.8 / 0.8 / 1.1
		Glu (mmol/L)	2.4 / 5.7 / 21.3	3.4 / 2.1 / 1.5
		Hb (g/dL)	7.7 / 14.7 / 21	1.7 / 0.9 / 0.8
Werfen GEM Chem STAT<br><br>Bench top analyzer	Measured: pH, PCO2, TCO2, lactate, Hte Hb, Na, K,Cl, iCa, Glu, ureaCrea	pH	7.13 / 7.36 / 7.51	0.11 / 0.06 / 0.33
		PCO2 (mmHg)	90.2 / 37.4 / 15.7	3.09 / 2.07 / 3.94
		Lactate (mmol/L)	8.4 / 5.1 / 1.7	2.33 / 3.46 / 4.92
		Na (mmol/L)	124.1 / 138.8 / 151.7	0.58 / 0.51 / 0.68
		K (mmol/L)	2.6 / 4.6 / 7.4	1.69 / 1.17 / 0.96
		Cl (mmol/L)	92.2 / 107.1 / 135.6	0.9 / 0.73 / 0.79
		iCa (mmol/L)	0.9 / 1.1 / 1.6	1.58 / 1.36 / 1.48
		Glu (mmol/L)	21.4 / 6.4 / 4.4	0.82 / 1.2 / 2.06
		Urea (mmol/L)	5.6 / 13.4 : 20.3	2.6 / 3.4 / 3.92
		Creat (µmol/L)	75.1 / 219.2 / 412.8	5.86 / 3.13 / 2.33
		Hematocrit (%)	22 / 42 / 67	2.49 / 0.37 / 0.57
Instrumentation laboratory Hemochron Signature Elite system.<br><br>Hand held analyzer	Hemochron Signature Elite Fresh Whole-Blood PT Cartridge<br>Hemochron Signature Elite Citrate PT Cartridge	INR	2.3 / 8.9	0.7 / 0.43* (*SD)
		TP	26 / 55	7.4 / 5

Sodium (Na), Potassium (K), Chloride (Cl), Glucose (Glu), Blood urea nitrogen (BUN/Urea), Hematocrit (Hct), Hemoglobin (Hb), Partial pressure of oxygen (PO2), Partial pressure of carbon dioxide (PCO2), Bicarbonate (HCO3), Total carbon dioxide (TCO2), Base excess (BE), Oxygen saturation (sO2), Ionized calcium (iCa), Creatinine (Crea), Anion gap (AnGap), Estimated glomerular filtration rate (eGFR), O_2Hb, oxyhemoglobin; COHb,carboxyhemoglobin; MetHb, methemoglobin; HHb, deoxyhemoglobin; HbF, fetal hemoglobin tBil, total bilirubin;

Table S1 references

Abbot istat

2. Steinfelder-Visscher, J.; Teerenstra, S. Evaluation of the I-STAT Point-of-Care Analyzer in Critically Ill Adult Patients. *J. Extra Corpor. Technol.* **2008**, *40*, 57–60.
4. Lee, M.-Y.; Lim, S.-F.; Lam, L. Evaluation of the I-STAT Alinity Point-of-Care Analyzer. *Point Care* **2019**, *18*, 48–55. https://doi.org/10.1097/POC.0000000000000190.
27. Vranken, N.P.A.; Theelen, A.J.A.J.; Orbons, T.; Barenbrug, P.J.C.; Weerwind, P.W. Point-of-Care Measurement of Kaolin Activated Clotting Time during Cardiopulmonary Bypass: A Single Sample Comparison between ACT Plus and i-STAT. *J. Extra Corpor. Technol.* **2021**, *53*, 57–61. https://doi.org/10.1182/ject-2000046.
28. Papadea, C.; Foster, J.; Grant, S.; Ballard, S.A.; Iv, J.C.C.; Michael, W.; Purohit, D.M. Evaluation of the I-STAT Portable Clinical Analyzer for Point-of- Care Blood Testing in the Intensive Care Units of a University Children's Hospital. *Ann. Clin. Lab. Sci.* **2002**, *32*, 231–243.
29. Füzéry, A.K.; Elian, F.A.; Kost, G.J. A Review of Temperature-Related Challenges and Solutions for the Abbott i-STAT and Siemens Healthineers Epoc Devices. *Clin. Biochem.* **2022**. https://doi.org/10.1016/j.clinbiochem.2022.08.013

Nova Stat Profile Prime Plus :

30. Jawa, A.; Motara, F.; Moolla, M.; Laher, A.E. A Comparative Assessment of the Nova Stat Profile Prime Plus® Critical Care Analyzer. *Cureus* **2020**, doi:10.7759/cureus.9932.

Nova Stat Sensor creatinine:

21. Snaith, B.; Harris, M.A.; Shinkins, B.; Jordaan, M.; Messenger, M.; Lewington, A. Point-of-Care Creatinine Testing for Kidney Function Measurement Prior to Contrast-Enhanced Diagnostic Imaging: Evaluation of the Performance of Three Systems for Clinical Utility. *Clin. Chem. Lab. Med.* **2018**, *56*, 1269–1276. https://doi.org/10.1515/cclm-2018-0128.

Nova Stat Strip Lactate

31. Tolan, N.V.; Wockenfus, A.M.; Koch, C.D.; Crews, B.O.; Dietzen, D.J.; Karon, B.S. Analytical Performance of Three Whole Blood Point-of-Care Lactate Devices Compared to Plasma Lactate Comparison Methods and a Flow-Injection Mass Spectrometry Method. *Clin. Biochem.* **2017**, *50*, 168–173, doi:10.1016/j.clinbiochem.2016.11.004

Radiometer ABL90 FLEX Plus

7. Bargnoux, A.-S.; Kuster, N.; Sutra, T.; Laroche, L.; Rodriguez, A.; Morena, M.; Chenine, L.; Chalabi, L.; Dupuy, A.-M.; Badiou, S.; et al. Evaluation of a New Point-of-Care Testing for Creatinine and Urea Measurement. *Scand J. Clin. Lab. Investig.* **2021**, *81*, 290–297. https://doi.org/10.1080/00365513.2021.1914344.
12. Kuster, N.; Bargnoux, A.S.; Badiou, S.; Dupuy, A.-M.; Piéroni, L.; Cristol, J.P. Multilevel Qualification of a Large Set of Blood Gas Analyzers: Which Performance Goals? *Clin. Biochem.* **2019**, *74*, 47–53. https://doi.org/10.1016/j.clinbiochem.2019.09.005.

Roche Cobas b 123

32. De Koninck, A.-S.; De Decker, K.; Van Bocxlaer, J.; Meeus, P.; Van Hoovels, L. Analytical Performance Evaluation of Four Cartridge-Type Blood Gas Analyzers. *Clin. Chem. Lab. Med.* **2012**, 50, doi:10.1515/cclm-2011-0685

Roche CoaguCheck Pro II

33. Nam, M.; Hur, M.; Kim, H.; Yoon, S.; Lee, S.; Shin, S.; Moon, H.; Yun, Y. Performance evaluation of coaguchek pro II in comparison with coaguchek XS plus and sta-r Max using a sta-neoplastine CI plus. *Int. J. Lab. Hematol.* **2021**, *43*, 1191–1197, doi:10.1111/ijlh.13466.

Siemens Rapid Point

34. Mion, M.M.; Bragato, G.; Zaninotto, M.; Alessandroni, J.; Bernardini, S.; Plebani, M. Analytical Performance Evaluation of the New GEM® PremierTM 5000 Analyzer in Comparison to the GEM® PremierTM 4000 and the RapidPoint® 405 Systems. *Clin. Chim. Acta* **2018**, *486*, 313–319, doi:10.1016/j.cca.2018.08.019.

Siemens epoc® Blood Analysis System

35. Danielsen, J.S.; Voigt, C.T.; Koefod, H.; Jacobsen, K.K. Assessment of the Analytical Performance of 14 Analytes Using the Epoc® Blood Analysis System. *Int. J. Biomed. Lab. Sci.* **2022**, *11*, 104–113.

Siemens Xprecia Stride™ Coagulation Analyzer

36. Piacenza, F.; Galeazzi, R.; Cardelli, M.; Moroni, F.; Provinciali, M.; Pierpaoli, E.; Giovagnetti, S.; Appolloni, S.; Marchegiani, F. Precision and Accuracy of the New XPrecia Stride Mobile Coagulometer. *Thromb. Res.* **2017**, 156, 51–53, doi:10.1016/j.thromres.2017.05.032.

Werfen GEM Chem 5000

37. Oyaert, M.; Van Maerken, T.; Bridts, S.; Van Loon, S.; Laverge, H.; Stove, V. Analytical and Pre-Analytical Performance Characteristics of a Novel Cartridge-Type Blood Gas Analyzer for Point-of-Care and Laboratory Testing. *Clin. Biochem.* **2018**, *53*, 116–126, doi:10.1016/j.clinbiochem.2018.01.007.

Werfen GEM Chem STAT

38. García-Fernández, A.E.; Barquín, R.; Martínez, M.; Ferrer, R.; Casis, E.; Xu, C. Performance Evaluation of a Point of Care Cartridge of the New GEM Premier ChemSTAT Analyzer. *Pract. Lab. Med.* **2022**, *31*, e00297, doi:10.1016/j.plabm.2022.e00297.

Instrumentation laboratory Hemochron Signature Elite system

39. Toben, B.; Martin, M. Rapid Assessment of Coagulation at the Point of Care With the Hemochron Signature Elite System. *Point Care* **2020**, *19*, 116, doi:10.1097/POC.0000000000000216.
